# Frequent mutation of the *FOXA1* untranslated region in prostate cancer

**DOI:** 10.1038/s42003-018-0128-1

**Published:** 2018-08-24

**Authors:** Matti Annala, Sinja Taavitsainen, Gillian Vandekerkhove, Jack V. W. Bacon, Kevin Beja, Kim N. Chi, Matti Nykter, Alexander W. Wyatt

**Affiliations:** 1 Faculty of Medicine and Life Sciences and Biomeditech Institute, University of Tampere, FI-33520 Tampere, Finland; 2 Vancouver Prostate Centre, Department of Urologic Sciences, University of British Columbia, Vancouver, BC V6H 3Z6 Canada; 3 Department of Medical Oncology, British Columbia Cancer Agency, Vancouver, BC V5Z 1G1 Canada

## Abstract

Prostate cancer has a low somatic mutation rate but non-coding regions remain underexplored. We sequenced the untranslated regions (UTRs) of 72 established driver genes in 428 patients with metastatic prostate cancer and identified *FOXA1* 3′-UTR mutations in 12% of patients. The mutations were predominantly insertions or deletions, covered the entire UTR without motif enrichment, and were not detected in other cancers. *FOXA1* lies in head-on orientation with the androgen-regulated non-coding gene *AL121790.1*, resulting in strong prostate lineage-specific bidirectional transcription across the *FOXA1* 3′-UTR. This suggests transcriptional activity as a cause for the localized hypermutation. The indel-dominant pattern of somatic mutation extends into the *FOXA1* coding region, where it is shaped by clonal selection to yield a cluster of non-frameshift indels inside the forkhead domain. Somatic *FOXA1* 3′-UTR mutations may prove useful for diagnostic and screening approaches, given their high frequency and lineage specificity.

## Abstract (web summary)

Matti Annala et al. report the recurrence of new *FOXA1* mutations in prostate cancer. These *FOXA1* mutations in 3’ untranslated region may prove useful as diagnostic markers for prostate cancer.

## Introduction

Prostate cancer is the second leading cause of cancer-related death in men, and has a well-described somatic coding genome defined by distinct molecular subclasses and high heterogeneity^1^. The mutation rate of prostate cancer is relatively low (0.9 and 4.4 mutations per Mb in primary and metastatic disease, respectively)^2,3^ and is characterized by an age-related CG>TG mutational signature^4^. Conversely, chromosomal rearrangements (e.g., activating fusions involving ETS family transcription factors) and copy number alterations (e.g., chromosome 8p loss and 8q gain) are highly prevalent^2^. Nevertheless, several genes including *SPOP*, *FOXA1*, and *TP53*, are recurrently mutated and considered to be drivers of tumorigenesis and/or progression^5^.

The majority of published prostate cancer studies have leveraged whole-exome sequencing approaches that do not capture the untranslated regions of protein coding genes. Although whole-genome sequencing is beginning to reveal deep insight into the non-coding cancer genome^6–9^, and has led to the discovery of several new cancer driving mechanisms^10–12^, the role of regulatory region mutations in localized and metastatic prostate cancer remains underexplored. To date, no recurrent mutations have been identified outside coding regions in prostate cancer.

In recent years, genomic profiling of lethal metastatic disease in advanced cancer patients has become more practical through the use of liquid biopsies. Cell-free circulating tumor DNA (ctDNA) is highly abundant in the plasma of metastatic castration-resistant prostate cancer (mCRPC) patients^13–15^. We have demonstrated that somatic mutation and copy number profiles for prostate cancer driver genes are strongly concordant between matched ctDNA and metastatic tissue specimens from mCRPC patients^16^. In a recent study of ctDNA-positive samples from 115 mCRPC patients we observed mutations within the UTRs of several prostate cancer driver genes, including *FOXA1* and the androgen receptor (*AR*)^15^. We hypothesized that some prostate cancer associated genes may exhibit an elevated UTR mutation rate. In this study, we report mutation frequencies in the UTRs of 72 known prostate cancer driver genes in 428 men with mCRPC, and identify an indel-dominated pattern of somatic mutation encompassing the entire *FOXA1* 3′-UTR and C-terminal.

## Results

### Low mutation rate in UTRs of prostate cancer driver genes

**Fig. 1:**
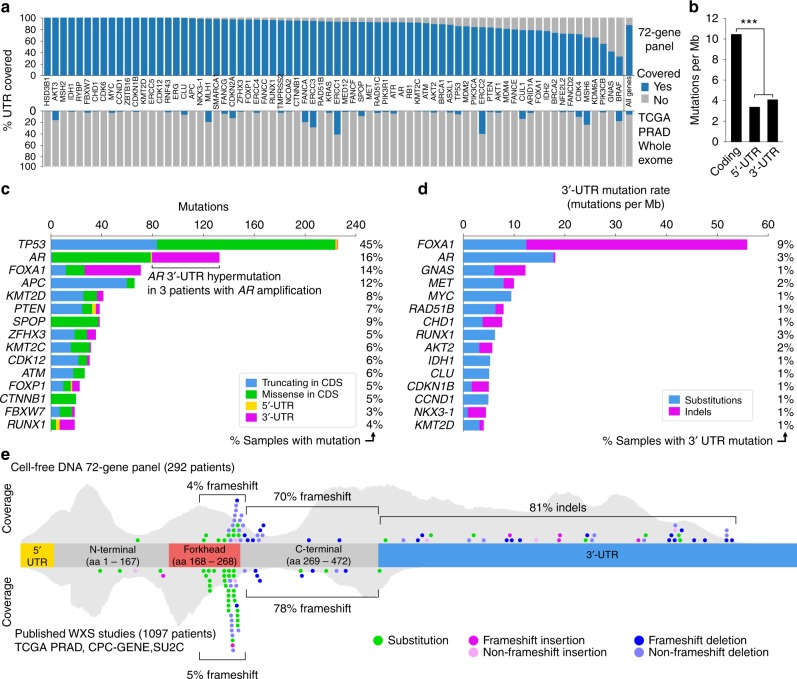
Somatic mutation rates in the untranslated regions of prostate cancer driver genes. **a** Comparison of untranslated region coverage between our 72-gene panel and the Agilent SureSelect Human All Exon panel used by the Cancer Genome Atlas prostate adenocarcinoma (TCGA PRAD) working group. Coverage threshold was 200× for the 72-gene panel (sufficient for mutation detection from ∼10% ctDNA sample) and 50× for the Agilent SureSelect Human All Exon panel (sufficient for mutation detection from tissue with ∼30% cancer fraction). Incomplete coverage of *BRAF*, *GNAS*, and *PIK3CB* UTRs was due to differences in annotated UTR length between RefSeq (used for panel design) and Ensembl (used in this study). **b** Comparison of somatic mutation rate in the coding vs untranslated regions covered by our 72-gene panel. Mutation rate was defined as the total number of somatic mutations, divided by the number of genomic positions with >200× coverage (in megabases), divided by the number of cfDNA samples with ctDNA >2%. **c** Bar plot showing 15 genes with the highest number of somatic mutations, broken down by mutation type and region. Percentage of samples carrying one of the included mutation types is shown on the right. **d** Bar plot showing 15 genes with the highest 3′-UTR mutation rate. Mutation count was normalized by number of ctDNA-positive samples (*n* = 439) and 3′-UTR length in megabases. Percentage of samples carrying a 3′-UTR mutation is shown on the right. **e** Distribution of somatic mutations along the *FOXA1* exonic regions, in our cohort (top) and in published whole-exome sequencing cohorts (bottom). For consistency with the coding region, indels in the 3′-UTR were also colored based on length, although they cannot result in frameshifts. Gray silhouettes indicate sequencing coverage

We performed targeted sequencing across all exons of 72 prostate cancer driver genes in 712 plasma cell-free DNA (cfDNA) samples from 428 mCRPC patients (Supplementary Data 1). Circulating tumor DNA fraction was above 2% of total cfDNA in 439/712 (62%) samples (290/428 patients, 68%). Samples with ctDNA fractions below 2% were not included in further analyses due to reduced sensitivity for mutation detection^15^. Across the 72 genes in our panel, we achieved ≥200× sequencing depth for 94% and 70% of positions within annotated 3′- and 5′-UTRs, respectively (Fig. 1a, Supplementary Fig. 1, Supplementary Data 2 and 3). Median sequencing depth inside targeted regions was 751× (IQR 616× to 899×). At this sequencing depth, we achieve >90% sensitivity for detecting somatic mutations at allele fractions above 3.5%, as previously shown^15^.

A total of 1945 somatic mutations were detected in the 439 ctDNA-positive samples (Supplementary Data 1). Of these 1945 mutations, 992 (51%) were in coding regions, 271 (14%) were in UTRs, and 682 (35%) were in introns or intergenic regions. The average somatic mutation rate in UTRs was significantly lower than for coding regions (4.0 vs 10.5, *p* < 10^−16^, binomial test) and was similar between 5′- and 3′-UTRs (Fig. 1b), but varied between genes. The high mutation rate in coding regions was expected since our panel targets recurrently mutated prostate cancer genes. The fraction of substitution vs indel mutations was similar in UTRs and coding regions (73% vs 68% substitutions, *p* = 0.095, Fisher’s exact test). In line with large published prostate cancer cohorts^2,3,15^, the most commonly mutated genes were *TP53*, *AR*, *FOXA1*, *APC*, *KMT2D, SPOP*, and *PTEN*, primarily driven by missense and truncating mutations inside the coding region (Fig. 1c).

### The *FOXA1* 3′-UTR displays a high rate of indel mutations

The highest number of non-coding mutations was observed in the *FOXA1* 3′-UTR, which harbored 37 somatic mutations in 34/290 (12%) mCRPC patients (Fig. 1c, Supplementary Data 4). The androgen receptor (*AR*) 3′-UTR harbored the second highest number of non-coding mutations, but this was driven by 3 patients that carried *AR* gene amplification together with *AR* 3′-UTR hypermutation (Supplementary Data 4). No other UTR in our panel was mutated in more than 10 patients, suggesting that UTR somatic mutation is not a common mechanism of driver gene deregulation in prostate cancer. The *FOXA1* 3′-UTR is 1814 bp in length, slightly larger than the median of other 3′-UTRs in our panel (1344 bp, range 0–10.2 kb). However, when controlling for UTR length, *FOXA1* still displayed three times as many mutations per kb than any other 3′-UTR (Fig. 1d). Among the 71 other genes included in our panel, a total of 141 somatic 3′-UTR mutations were detected, only 19 (13%) of which were indels. In contrast, *FOXA1* 3′-UTR mutations were predominantly deletions (62%) or insertions (19%) (81% vs 13% indels, *p* = 1.6e−14, Fisher’s exact test) (Fig. 1d, Supplementary Data 4). The *FOXA1* 3′-UTR deletions ranged in size between 1 and 89 bp (median 8 bp), and insertions between 1 and 99 bp (median 18 bp). With the exception of a 3 bp deletion inside an AACAAC microsatellite (identified in both PC-225 and PC-074), all 3′-UTR mutations were unique to a single patient. Mutations were heterozygous in all evaluable cases. Sanger sequencing confirmed the presence of high allelic frequency *FOXA1* 3′-UTR indel mutations in 5/5 cfDNA samples that were tested (Supplementary Fig. 2). As further validation, we obtained diagnostic tumor tissue from two patients (PC-225 and PC-235) whose cfDNA showed FOXA1 3′-UTR mutations. In both cases the same mutations were detected in tissue by targeted and Sanger sequencing (Supplementary Figs. 2−4, Supplementary Data 5). Finally, to confirm FOXA1 3′-UTR mutations in whole-genome sequencing data, we studied a published cohort of 52 metastatic tissue samples from 10 prostate cancer patients^17^, and identified FOXA1 3′-UTR mutations in two patients. The mutations were present in all metastatic tissue from these patients (Supplementary Data 5, Supplementary Figs. 5, 6).

### The indel pattern extends into the FOXA1 coding region

The *FOXA1* coding region is mutated in 5–10% of prostate tumors^2,3,5,18^, with over half of reported mutations falling within or near the forkhead domain (Fig. 1e). These mutations are presumed to confer a selective advantage via altered DNA binding specificity^19^. However, occasional indel mutations have also been reported downstream of the forkhead domain, in the C-terminal transactivation domain^2^. The impact of these (often truncating) mutations is unclear. In our cohort, 34/290 patients (12%) harbored mutations within the FOXA1 coding region, including 23 mutations that fell within or near the forkhead domain, consistent with published studies (Fig. 1e)^2,5^. In total, 57/290 (20%) patients in our cohort harbored at least one *FOXA1* coding region or UTR mutation.

**Fig. 2:**
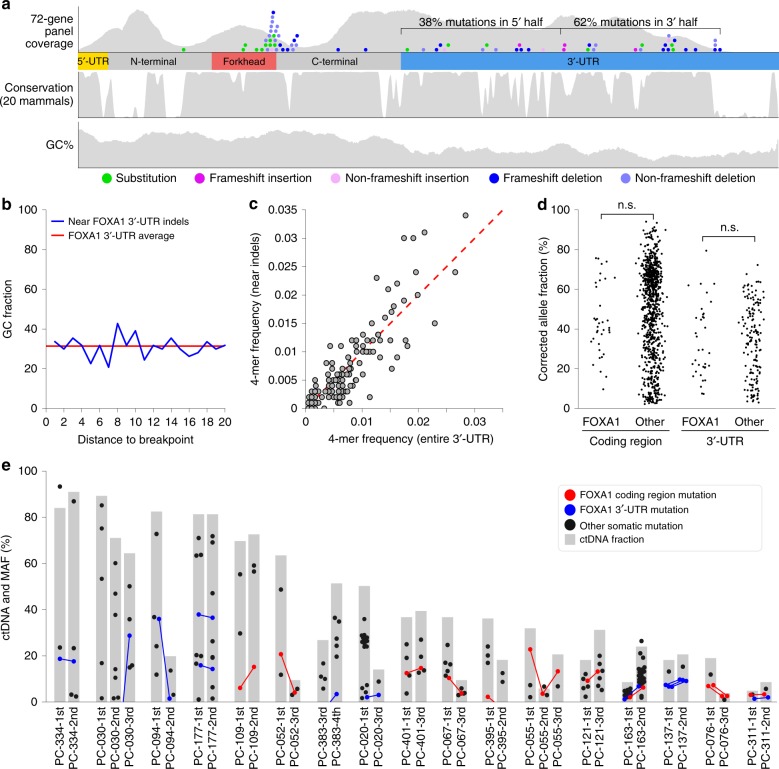
A localized hypermutation process affects the entire *FOXA1* 3′-UTR. **a** Tracks showing the 72-gene panel sequencing coverage, phylogenetic conservation score (PhastCons20, 20 mammal species), and GC percentage calculated using a 20 bp sliding window. **b** Average GC percentage at different distances around *FOXA1* 3′-UTR indel mutations. **c** Scatter plot showing the frequency of 4-mers within 20 bp neighborhoods around *FOXA1* 3′-UTR indel mutations (*y*-axis) and the frequency of 4-mers across the entire *FOXA1* 3′-UTR. **d** Beeswarm plot showing ctDNA%-corrected allele fractions of coding region and 3′-UTR mutations in the 439 cfDNA samples with ctDNA. Allele fractions are shown separately for *FOXA1* and the other 71 genes. **e** Evolution of FOXA1 mutation allele fractions across time. Each group of bars represents one of 17 patients that had multiple cfDNA timepoints with ctDNA > 5%. Heights of gray bars represent estimated ctDNA%. Dots represent somatic mutations, with *y*-axis position representing allele fraction. Lines connecting dots indicate that the same FOXA1 mutation was observed at multiple timepoints

Overlapping our mutation analysis with published coding region data revealed a clear pattern of indel mutations affecting both the 3′-UTR and the coding region as far as the forkhead domain (Fig. 1e). Mutation density was slightly higher at the 3′ end of the *FOXA1* 3′-UTR, and tapered off towards 5′ end and the coding region (Fig. 2a). Very few frameshift indels were observed in amino acids 1–272, only inframe events, suggesting negative selective pressure for alterations that truncate or inactivate the forkhead domain. Base composition within a 20 bp neighborhood around the breakpoints was consistent with the 3′-UTR average (Fig. 2b), and we did not identify any recurrent sequence motifs adjacent to the indel breakpoints (Fig. 2c). Of the 22 deletions inside the *FOXA1* 3′-UTR, 5 involved deletion of one repeat from a tandem repeat sequence (e.g. AATAATAAT->AATAAT deletion in PC-235), while 6/7 insertions inside the 3′-UTR were short-tandem duplications (Supplementary Data 6). Phylogenetic conservation scores around somatic mutations were in line with the 3′-UTR average (average 0.73 vs 0.65, *p* = 0.66, rank-sum test) (Fig. 2a). Taken together, these results suggest that the mutation process affects the entire 3′-UTR without enrichment at specific regions.

*FOXA1* 3′-UTR mutations were detected more frequently in patients that also carried mutations in the *FOXA1* coding region (32% vs 8%, *p* = 0.0007, Fisher’s exact test). We found no association between *FOXA1* 3′-UTR mutations and genomic alterations in other recurrently mutated genes (Supplementary Fig. 7).

### *FOXA1* UTR mutations are found in early stage prostate cancer

Mutations that arise late in cancer development are sometimes only present in a subset of ctDNA-releasing cancer cells, and can exhibit a lower mutant allele fraction in cfDNA. In the 72 genes captured by our panel, ctDNA%-corrected mutant allele fractions were generally lower in 3′-UTRs than in coding regions (0.34 vs 0.54, *p* = 1.1e−19, ranksum test), consistent with coding region mutations being subject to positive selection and loss-of-heterozygosity (Fig. 2d). The allele fractions of *FOXA1* 3′-UTR mutations, after correction for ctDNA%, were not significantly higher or lower than allele fractions of 3′-UTR mutations in other genes (*p* = 0.95) (Fig. 2d). The same was true of *FOXA1* coding region mutations (*p* = 0.09) (Fig. 2d). This suggests that *FOXA1* 3′-UTR mutations are not simply a late or predominantly subclonal event in prostate cancer. To investigate further, we examined nine mCRPC patients with *FOXA1* 3′-UTR mutations who provided multiple ctDNA-positive (ctDNA > 5%) specimens over their clinical time course. In seven of these patients, the same *FOXA1* 3′-UTR mutation was detected at all timepoints (Fig. 2e). In two patients (PC-030 and PC-383), the 3′-UTR mutation harboring clone rose to prominence late in their clinical time course, based on complete absence of supporting reads in earlier timepoints (Fig. 2e, Supplementary Fig. 8). Evidence for loss of *FOXA1* 3′-UTR mutation from ctDNA over time was not observed in any patients.

Secondly, we interrogated a published dataset (generated with the identical 72 gene panel) of matched diagnostic tissue biopsy and radical prostatectomy specimens from localized prostate cancer^20^. In this dataset, there were 33 patients with at least one somatic mutation within the 72 gene panel, and 3/33 (9%) harbored *FOXA1* 3′-UTR indel mutations. All three patients were positive for the same *FOXA1* 3′-UTR mutation in their diagnostic needle biopsy and in their radical prostatectomy specimen collected 4–6 months later (Supplementary Data 5). Together, these results suggest that *FOXA1* 3′-UTR indel mutations are prevalent in both primary and metastatic disease but do not show enrichment consistent with selection in advanced disease.

Consistent with a passenger role in prostate cancer cells, among the 202 mCRPC patients in our cohort with long term follow-up data^15^, presence of *FOXA1* 3′-UTR alterations did not influence time to progression on first-line abiraterone or enzalutamide therapy (5.6 vs 5.5 months, hazard ratio = 1.03, 95% CI 0.57–1.87, *p* = 0.92, univariate Cox proportional hazards model) or overall survival from mCRPC treatment initiation (16.6 vs 18.2 months, hazard ratio = 1.46, 95% CI 0.78–2.74, *p* = 0.24) (Supplementary Fig. 9, Supplementary Data 7).

### *FOXA1* UTR indels are not detected in other cancers

**Fig. 3:**
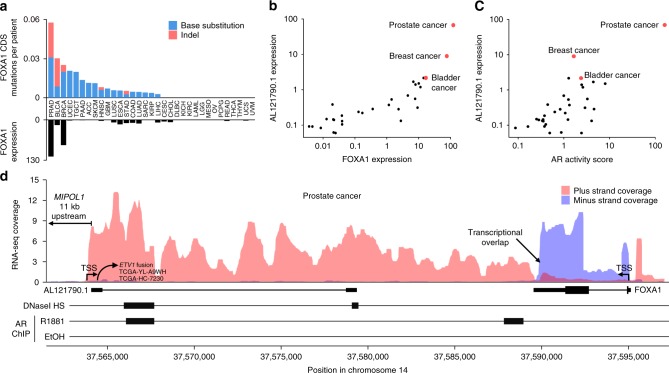
*FOXA1* 3′-UTR mutations are prostate cancer specific and associated with high bidirectional transcriptional activity. **a** Barplot showing the number and type of *FOXA1* mutations in 33 TCGA cancer types (data obtained from cBioPortal); ordered by mutation rate. Bottom barplot shows *FOXA1* expression level. **b** Scatter plot showing correlation between *FOXA1* and *AL121790.1* expression across 33 TCGA cancer types. **c** Scatter plot showing correlation between androgen receptor (AR) activity score and *AL121790.1* expression across 33 TCGA cancer types. **d** Genomic visualization illustrating transcriptional overlap between *FOXA1* and *AL121790.1*, inside the *FOXA1* 3′-UTR. Red and blue tracks show strand-specific RNA-seq coverage in prostate adenocarcinoma tissue. RNA-seq coverage was normalized by library size (reads per million, RPM). Transcription start sites (TSS) are indicated with arrows. At the bottom, AR ChIP-seq data from prostate cancer cell line LNCaP treated with synthetic androgen R1881 suggests presence of AR binding site 3 kb downstream of the *AL121790.1* TSS. Location of RNA-level fusion junction for *ETV1* fusions in the TCGA prostate adenocarcinoma cohort is also shown

*FOXA1* is only expressed in some adult tissues, with the highest levels observed in prostate tissue (Supplementary Fig. 10). Accordingly, prostate cancer exhibits higher *FOXA1* expression than other malignancies (Fig. 3a). Breast and bladder cancer express the second and third highest levels of *FOXA1*, respectively. Using a published bladder cancer focused approach^21^, we assessed the *FOXA1* mutation rate in 71 advanced bladder cancer patients. Although the bladder cancer panel achieved high sequencing depth throughout the FOXA1 3′-UTR (Supplementary Fig. 11), only one mutation (a base substitution) was identified among the 53/71 patients with sufficient ctDNA or cancer tissue cellularity (*p* = 0.04 for comparison with 33/290 in prostate cancer, Fisher’s exact test). Although we could not directly assess the *FOXA1* 3′-UTR in breast cancer samples, we note that somatic indels in the *FOXA1* coding region are significantly less frequent in breast cancer than prostate cancer (5/982 in TCGA breast invasive carcinomas vs 15/499 in TCGA prostate adenocarcinomas, *p* = 0.0002, Fisher’s exact test). Together these data suggest that bladder and breast cancer do not harbor the distinctive indel signature observed 3′ of the FOXA1 forkhead domain in prostate cancer.

### *FOXA1* UTR is subject to strong bidirectional transcription

Recurrent translocation of the ETS family gene *ETV1* to a region downstream of *FOXA1* on chromosome 14 has been observed in prostate cancer patients and cell lines^22,23^. Although the *MIPOL1* loci (the adjacent coding gene) is often described as the insertion point for these *ETV1* translocations, the 38 kb region between *MIPOL1* and *FOXA1* harbors the non-coding gene *AL121790.1* (also known as *ENSG00000258414* and *EST14*) that has been reported to exhibit androgen-regulated and prostate-specific expression^22^. Fusion of *AL121790.1* with *ETV1* is consistent with other ETS rearrangements in prostate cancer, which typically involve an AR driven promoter^1^. Analysis of RNA sequencing data from 33 cancer types revealed that *AL121790.1* expression is strongly correlated with *FOXA1* expression (Spearman correlation = 0.84) (Fig. 3b). Expression of *AL121790.1* was highest in prostate cancer, 8-fold higher than in breast cancer and 30-fold higher than in any of the other 31 cancer types. We also confirmed the correlation between *AL121790.1* and AR activity (Fig. 3c) and additionally performed AR chromatin immunoprecipitation sequencing (ChIP-seq) on the prostate cancer cell line LNCaP treated with R1881 (a synthetic testosterone analog). We identified an AR binding site within 2.8 kb of the *AL121790.1* promoter, supported by the presence of a canonical androgen responsive element motif^24^ (Fig. 3d). Interestingly, in the TCGA prostate adenocarcinoma dataset^2^, the two patients with fusions juxtaposing the *MIPOL1* locus with *ETV1* had RNA-level fusion junctions that matched exactly with the end of the first annotated exon of *AL121790.1* (Fig. 3d)—suggesting that this AR-regulated non-coding gene is a common 5′ fusion partner in *ETV1* gene rearrangements, where it drives *ETV1* overexpression through its highly active promoter.

To understand whether the mutation process extends downstream of the *FOXA1* 3′-UTR, we analyzed whole-genome sequencing data from the aforementioned cohort 10 metastatic prostate cancer patients and identified five somatic mutations in the 25 kb region between *AL121790.1* TSS and *FOXA1*, and no mutations in the non-transcribed 11 kb region between *MIPOL1* and the *AL121790.1* (Supplementary Data 5). This supports the hypothesis that the high mutation burden in this region is associated with transcription.

To further investigate a potential relationship between *AL121790.1* expression, *FOXA1* expression, and *FOXA1* 3′-UTR mutations, we studied published strand-specific RNA sequencing data from prostate adenocarcinoma tissue^25^. *AL121790.1* is in head-on orientation with *FOXA1*, and its RNA products overlap with the *FOXA1* 3′-UTR in prostate cancer cells (Fig. 3d). Published strand-specific RNA sequencing data from breast^26^ and bladder cancer (SRA project SRP103878) revealed no evidence for transcriptional overlap at the *FOXA1* 3′-UTR, due to low *AL121790.1* expression (Supplementary Fig. 12). Interestingly, the entire region spanning *AL121790.1* and *FOXA1* has been identified as a super-enhancer (dbSUPER:SE_33900) in various human cell types, with a topologically associated domain encompassing both *AL121790.1* and *FOXA1*, suggesting shared regulation. Ultimately, the strong bidirectional transcription reported here raises the possibility of head-on transcriptional collisions, transcriptional stalling, and replication-transcription collisions^27^, which may increase the potential for DNA damage in this region^28^.

## Discussion

Prostate cancer has a low somatic mutation rate in coding regions, and our data suggest this extends to the UTRs of known prostate cancer driver genes, with the notable exception of *FOXA1*. The previously unrecognized *FOXA1* 3′-UTR mutations reported here, together with established forkhead domain mutations, implicate *FOXA1* as the third most commonly mutated gene in advanced prostate cancer, after *TP53 and AR*. However, although FOXA1 forkhead domain mutations contribute to prostate cancer pathogenesis, the indel mutations we identified within the 3′-UTR and C-terminus appear to be passenger events. We did not observe evidence for their selection at different stages of disease progression, nor were they localized to any particular hotspots downstream of the forkhead domain.

The UTRs of coding genes are not captured in standard whole-exome sequencing protocols, including those used in TCGA efforts. Furthermore, although hundreds of localized prostate tumors have been subjected to whole genome sequencing^29^, no study has searched for UTRs with an elevated mutation rate to our knowledge, and the typical 15–30X whole-genome sequencing depth can pose a challenge for the discovery of novel mutations. Support for *FOXA1* 3′-UTR localized hypermutation in prostate cancer can be drawn from the following: (1) in all but one example the 3′-UTR mutations were unique to individual patients, yet where patients had multiple plasma or tissue collections performed, the same mutations were observed; (2) mutations were detected at similar frequencies in our plasma cfDNA and tumor tissue cohorts; (3) applying our capture-based targeted sequencing approach to plasma cfDNA from patients with metastatic bladder cancer yielded no *FOXA1* 3′-UTR indel mutations; (4) Sanger sequencing of 3′-UTR mutations with high variant allele frequency confirmed their presence in all tested samples; and (5) analysis of independent published whole-genome sequencing data from metastatic prostate cancer revealed *FOXA1* 3′-UTR mutations in 2/10 patients.

Patients with *FOXA1* coding region mutations had a significantly higher likelihood of carrying *FOXA1* 3′-UTR mutations, suggesting that the probability of accruing any mutation in this region varies between patients. The connection between *FOXA1* coding region and UTR mutations, together with the unusually high rate of non-frameshift indels in the FOXA1 forkhead domain, suggests that prostate cancer driver mutations in this domain arise due to a combination of a localized mutation process and clonal selection.

A recent study demonstrated that certain highly expressed and lineage-specific genes in lung, liver, stomach, and thyroid cancers harbor 3′-UTR indel hotspots^8^. In some genes, this apparent localized hypermutation extended beyond the 3′-UTR into the intergenic region. The mechanistic etiology of the hypermutation signature was unclear, but similar to the *FOXA1* indel mutations reported here, the 3′-UTR indel hotspots did not appear to be under selective pressure. *FOXA1* expression is lineage restricted, and very high in prostate cancer. However, *FOXA1* is also transcribed in head-to-head orientation with another highly expressed and lineage-specific gene, *AL121790.1*. Given that *AL121790.1* is AR regulated, the combination of high *FOXA1* and *AL121790.1* expression (overlapping within the *FOXA1* exonic region) is likely unique to the prostate lineage. It is well established that collisions between DNA replication and transcription machinery can trigger mutations^28^. Indeed, high levels of transcription have been associated with genomic damage^30^. When RNA polymerase II complexes collide head-to-head, they cannot bypass each other, transcription halts, and the complexes require removal from DNA via ubiquitination-directed proteolysis^27^. This can increase the number of stalled RNA polymerase complexes in the locus^27^, facilitating collisions with DNA replication. Unfortunately, our current data do not allow us to disambiguate the relative contributions of bidirectional transcription, high cumulative transcription, or other mechanisms towards the *FOXA1* mutation process. We also cannot rule out the possibility that *FOXA1* and *AL121790.1* are expressed in a coordinated manner so that the genes are never simultaneously transcribed from the same chromosome copy. Future studies should assess whether lineage-restricted genes in other cancers lie in head-to-head orientation, and whether regions subjected to high levels of bidirectional transcription exhibit elevated indel mutation rates.

Liquid biopsy strategies such as the one employed here rely on the detection of somatic mutations to confirm the presence and fraction of tumor DNA in the circulation. Additionally, assays applied in the diagnostic setting or for disease recurrence monitoring are augmented by detection of somatic variants (or combination of variants) that inform on the cancer type. For example, while *TP53* mutations can be found in many different cancers, SPOP MATH domain mutations are restricted to prostate and endometrial cancers. In cancers with low mutation rates (such as prostate cancer), gene panels must span a large genomic territory to increase the probability of detecting informative somatic variants. We posit that the *FOXA1* 3′-UTR represents a useful region for liquid biopsy gene panels, since any panel spanning this region will capture ~20% of patients and the detection of UTR indels can help confirm prostate cancer origin for the ctDNA. Furthermore, the frequency of *FOXA1* locus mutations reported in this study may actually be underestimated due to reduced mutation calling sensitivity in samples with <5% ctDNA^15^.

In addition to recurrent *FOXA1* forkhead domain mutations, the 14q21.1 region is also a recurrent partner in *ETV1*-activating structural rearrangements in prostate cancer. In TCGA prostate adenocarcinoma samples, these fusions juxtapose the first exon of *AL121790.1* with *ETV1*, turning *ETV1* into an AR-regulated gene with high expression, as has been demonstrated in earlier studies^22,23^. This implicates the long non-coding gene *AL121790.1* as a facilitator in two distinct prostate cancer driving mechanisms (*FOXA1* mutations and *ETV1* fusions).

## Methods

### Study design and patients

Plasma cell free DNA samples from 428 mCRPC patients and 71 bladder cancer patients were sequenced as part of the genitourinary cancers liquid biopsy biobank program at the Vancouver Prostate Center, University of British Columbia and the British Columbia Cancer Agency. Approval for collection and genomic profiling of patient samples was granted by the University of British Columbia Research Ethics Board (certificate numbers H18-00944, H14-00738, H16-00934, and H09-01628). The study was conducted in accordance with the Declaration of Helsinki, and written informed consent was obtained from all participants prior to enrollment.

### Target capture and sequencing

A custom NimbleGen SeqCap EZ Choice target capture panel was used to capture the coding regions of 72 genes (Supplementary Data 1). For each sample, 10–100 ng of DNA was used for library preparation. White blood cell gDNA samples were sheared into 180 bp fragments with a Covaris focused-ultrasonicator. A-tailing, end repair, Illumina-compatible adapter ligation and PCR amplification (between 12 and 17 cycles) was performed. Library quantification was carried out with the NanoDrop spectrophotometer, and each library was run on an ethidium bromide gel to confirm success. Up to 25 purified sample libraries at a time were multiplexed to obtain single pools with a combined mass of 1 μg, allowing a minimum 40 ng input for each sample library. These pools were hybridized to the capture panel for a minimum of 16 h at 47 °C. The subsequent wash, recovery, and amplification of the captured regions was performed according to the NimbleGen SeqCap EZ system protocols. Final libraries were purified with Agencourt AMPure beads and quantitated using either the KAPA qPCR kit, or the Qubit 2.0 Fluorometer (Life Technologies) and Qubit dsDNA HS Assay Kit. Pools were diluted to 20 pM, and were sequenced on Illumina MiSeq (V3 600 cycle kit) or HiSeq 2500 (V4 250 cycle kit) machines.

### Sequence alignment and quality control

Paired-end reads were aligned against the hg38 reference genome using Bowtie-2.3.0^31^. Optical and PCR duplicates were removed using samblaster-0.1.24^32^. Adapters were trimmed in paired mode using cutadapt-1.11^33^. Low-quality read tails (smoothed baseq < 30) were trimmed using an in-house algorithm. cfDNA/WBC sample pairings were verified based on SNP genotypes.

### Analysis of somatic mutations

Somatic mutations were called in cfDNA samples by searching for variants with an alternate allele fraction of at least 1%, and at least 10 supporting reads. Additionally, the allele fraction was required to be 25 times higher than the background error rate (i.e., the average allele fraction across all WBC samples), and 3 times higher than the allele fraction in the paired WBC sample. The paired WBC sample was required to have at least 20 reads covering the site. Protein-level consequences of variants were predicted using ANNOVAR^34^. Somatic mutation analysis for bladder cancer cfDNA samples was carried out using the same methodology.

To search for large indels, unaligned reads from each sample were split into two 30 bp anchors (from the 5′ and 3′ ends of the read) and aligned to the hg38 genome. Discordant anchor pairs were grouped by position and breakpoint signature. Duplicate reads arising from the same original cfDNA fragment were discarded based on read IDs and read start positions. Long indel candidates supported by three or more unique cfDNA fragments were manually curated using IGV and BLAT. Indels longer than 500 bp were excluded from analysis to maintain focus on localized mutational processes.

For each of the 72 genes captured by our panel, we determined the coordinates of its 5′ and 3′ untranslated regions based on annotations from Ensembl 90. RNA sequencing data from TCGA prostate adenocarcinoma samples was used to determine the predominantly expressed splice variant of each gene. UTR mutations were not allowed to overlap any annotated CDS for the gene in any of its splice variants.

### Analysis of gene expression across cancer types

RNA sequencing data for 165 cancer samples encompassing 33 cancer types (5 cancer samples from each cancer type) was downloaded from NCI Genomic Data Commons (GDC) Data Portal in BAM format. RNA sequencing reads aligning to *FOXA1* neighbor genes (*FOXA1*, *AL121790.1*, *MIPOL1*) and seven AR-regulated genes (*TMPRSS2*, *KLK2*, *KLK3*, *SLC45A3*, *FKBP5*, *NKX3-1*, *ACSL3*) were counted using Subread^35^ and converted to RPKM units by normalizing with transcript length and library size. AR activity score was calculated as the median expression of the seven AR-regulated genes.

### Code availability

Computer code for analyses performed in this study is available in the Github repository https://github.com/annalam/foxa1-utr-manuscript-code. Code for structural rearrangement analysis is available in Github repository https://github.com/annalam/breakfast. Code for FASTQ/BAM file manipulation is available in Github repository https://github.com/annalam/seqkit.

### Data availability

De-identified sequencing data was deposited to the European Genome-phenome Archive (EGA) under study identifier EGAS00001003113 and is available under standard EGA controlled release. Patients signed informed consent for their blood specimens to be used for future cancer research purposes.

## Electronic supplementary material

Supplementary Information

Description of Additional Supplementary Information

Supplementary Dataset 1

Supplementary Dataset 2

Supplementary Dataset 3

Supplementary Dataset 4

Supplementary Dataset 5

Supplementary Dataset 6

Supplementary Dataset 7
