# Supplementary material for: Frequent mutation of the FOXA1 untranslated region in prostate cancer
Source: Commun Biol. 2018 Aug 24;1:122. doi: 10.1038/s42003-018-0128-1 (PMC6123809; doi:10.1038/s42003-018-0128-1)
Supplement: Supplementary file 1 — Supplementary Information [file 42003_2018_128_MOESM1_ESM.pdf]

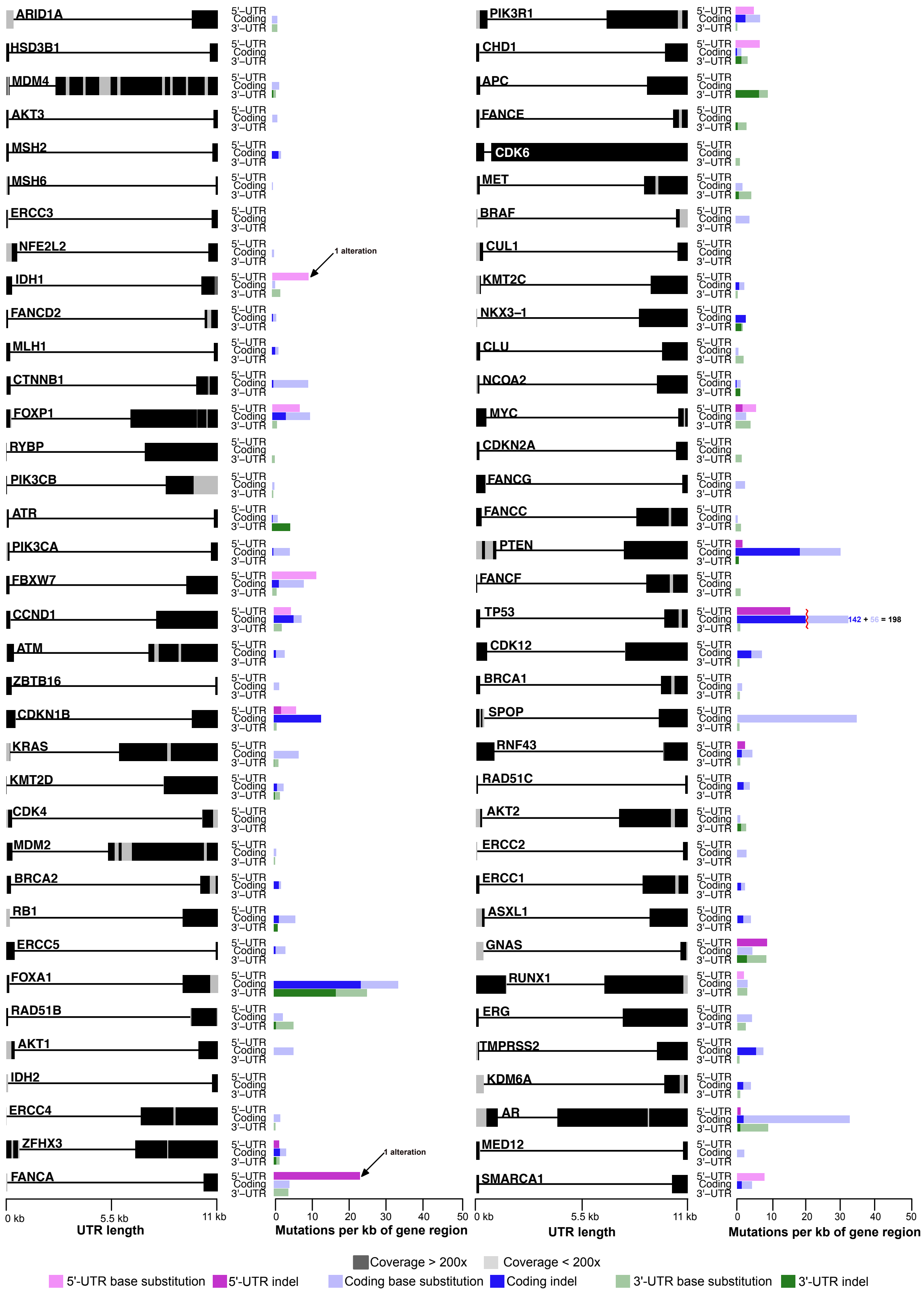

Supplementary Figure 1. Length and sequencing coverage of the untranslated regions of all 72 targeted genes. Each gene is depicted as a line, with rectangles on the left and right indicating the lengths of the 5'-UTR and 3'-UTR, respectively. Black segments indicate regions with coverage ≥ 200x coverage. Gray segments represent low coverage regions. Genes are sorted by chromosome.

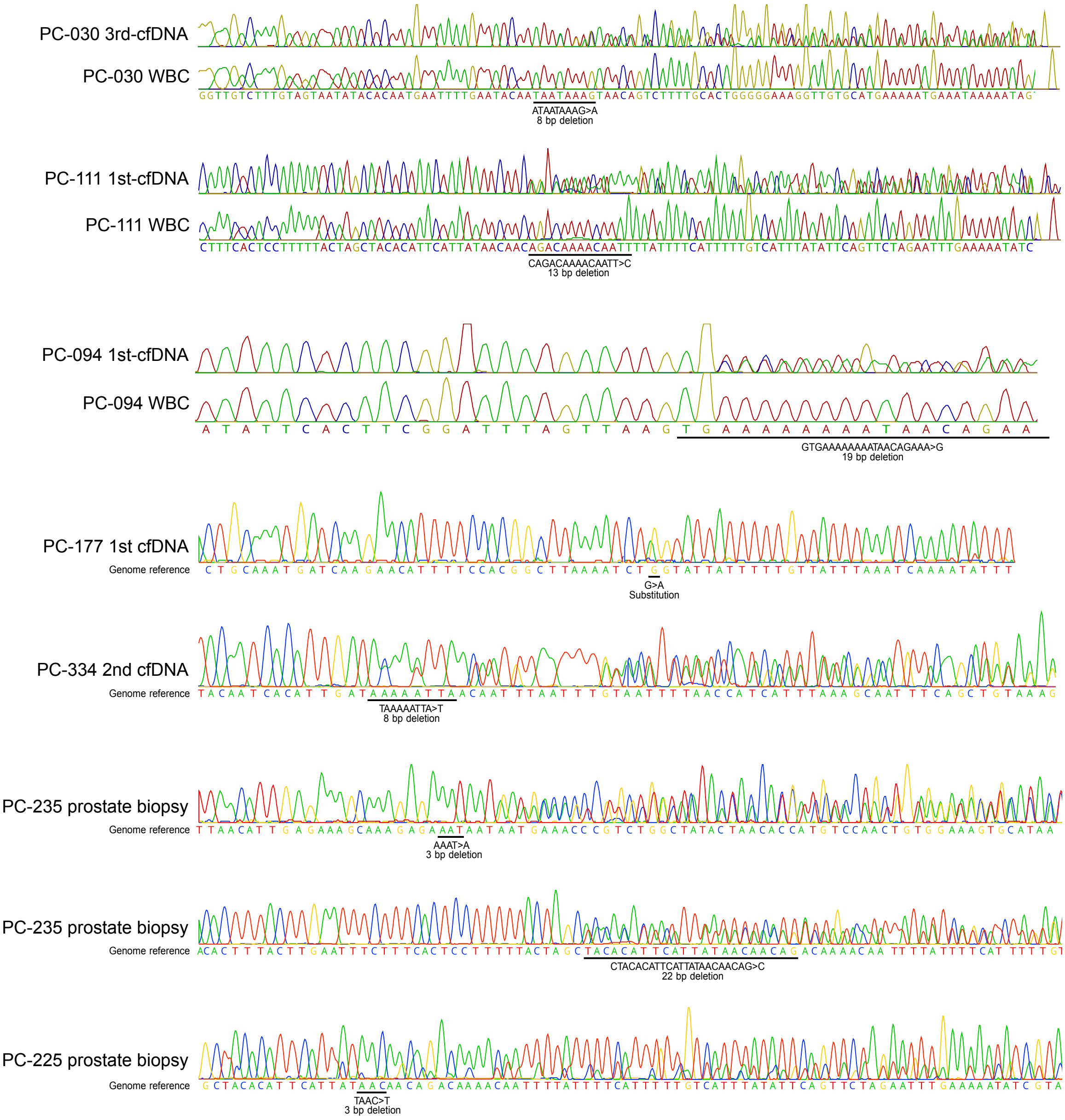

Supplementary Figure 2. Validation of seven deletions and one substitution in the *FOXA1* 3'-UTR using Sanger sequencing. The somatic mutations identified in targeted sequencing are indicated with black underlines.

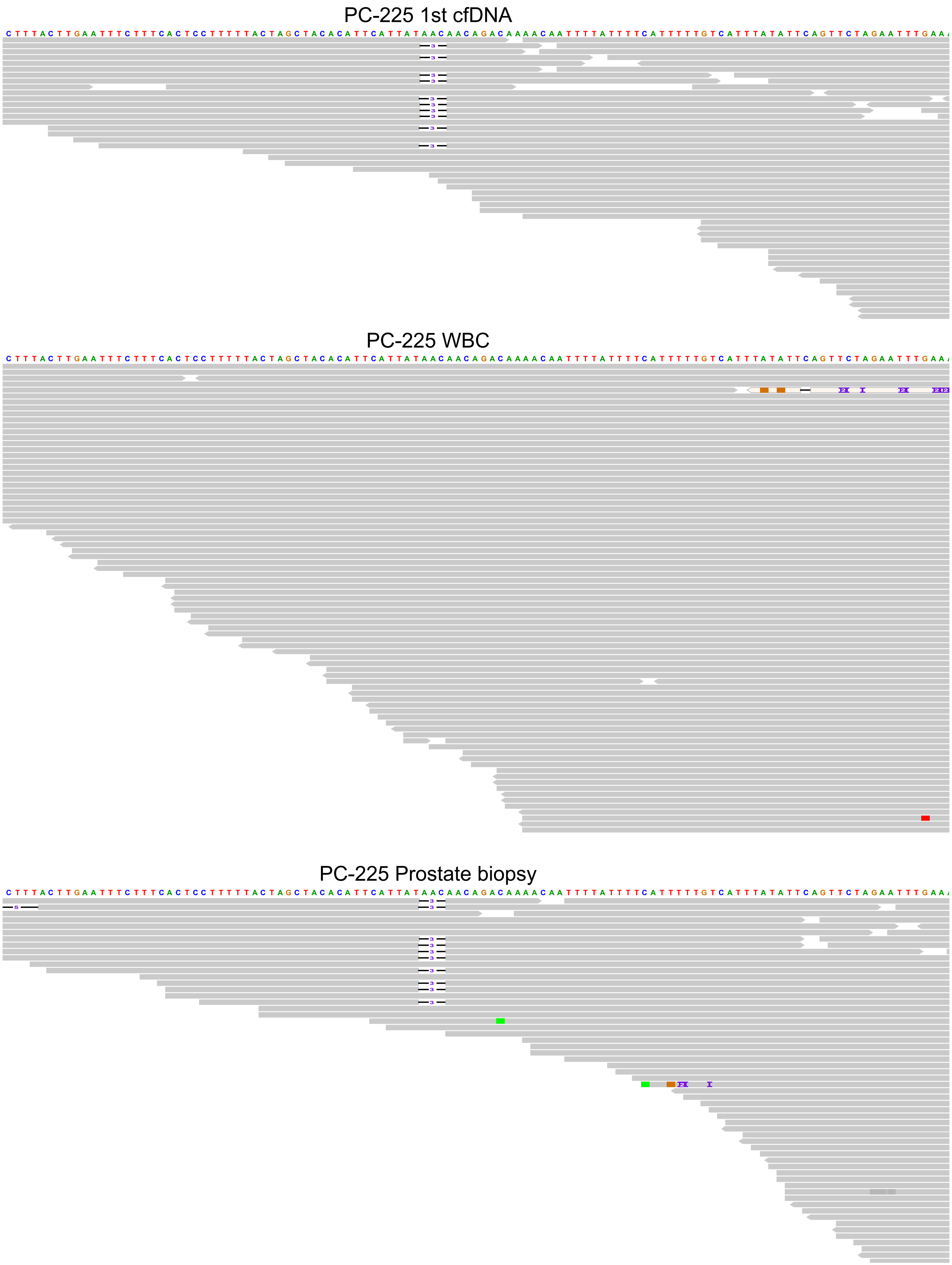

Supplementary Figure 3. Integrative Genomics Viewer (IGV) visualization of the reads supporting a somatic FOXA1 3'-UTR mutation in PC-225 cfDNA and tissue samples.

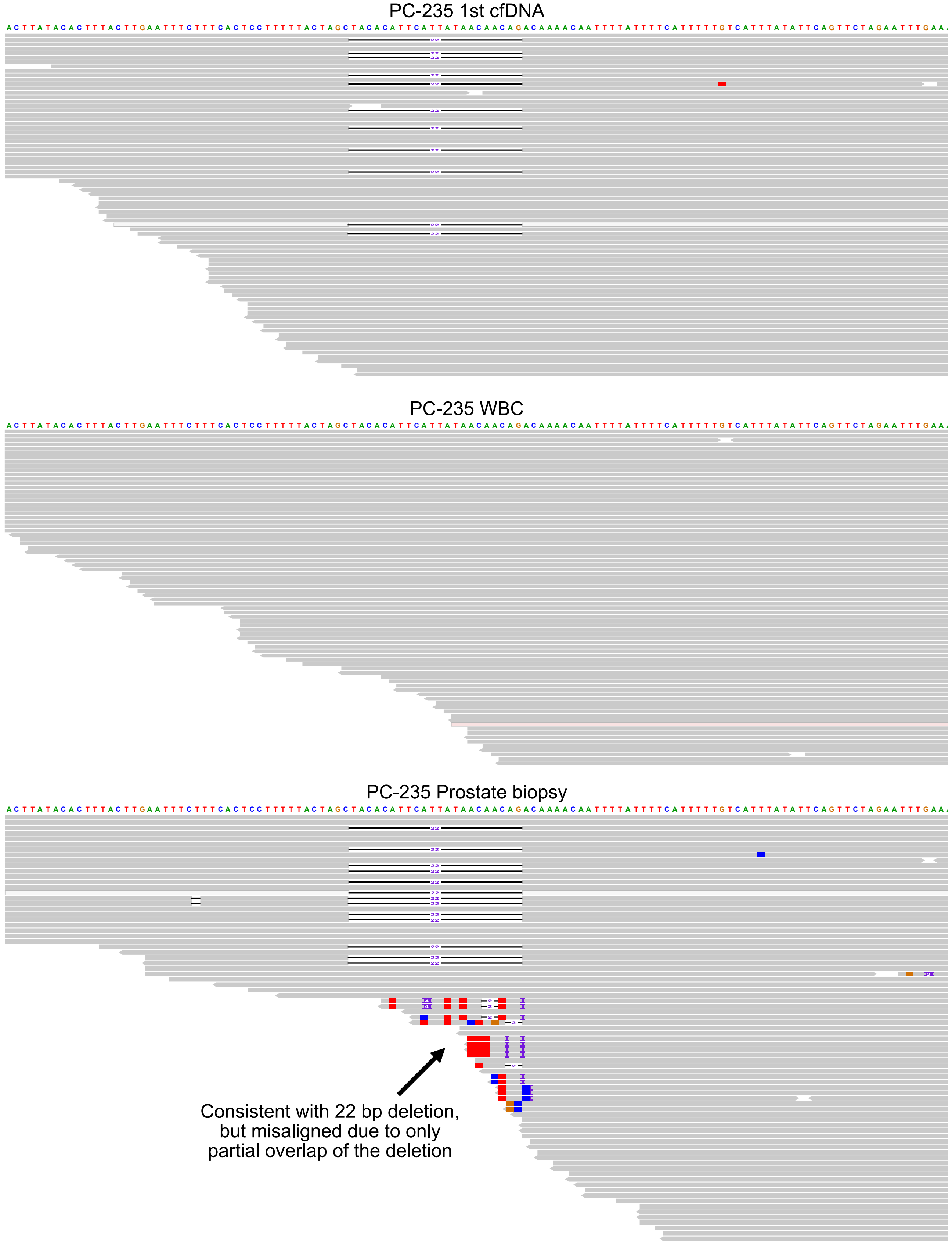

Supplementary Figure 4. Integrative Genomics Viewer (IGV) visualization of the reads supporting a somatic FOXA1 3'-UTR mutation in PC-235 cfDNA and tissue samples.

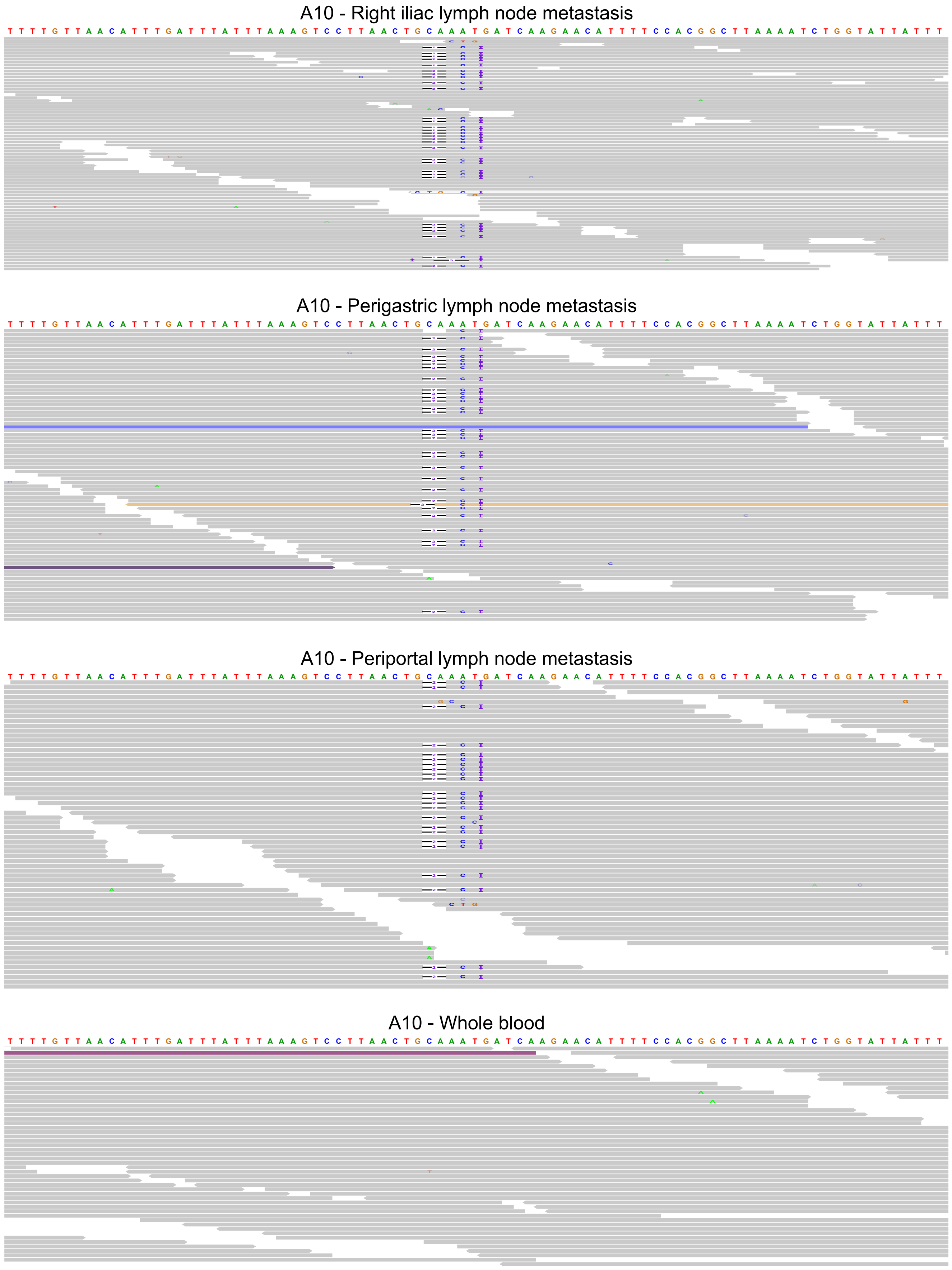

Supplementary Figure 5. Integrative Genomics Viewer (IGV) visualization of the reads supporting a somatic FOXA1 3'-UTR mutation in metastatic tissues of patient A10.

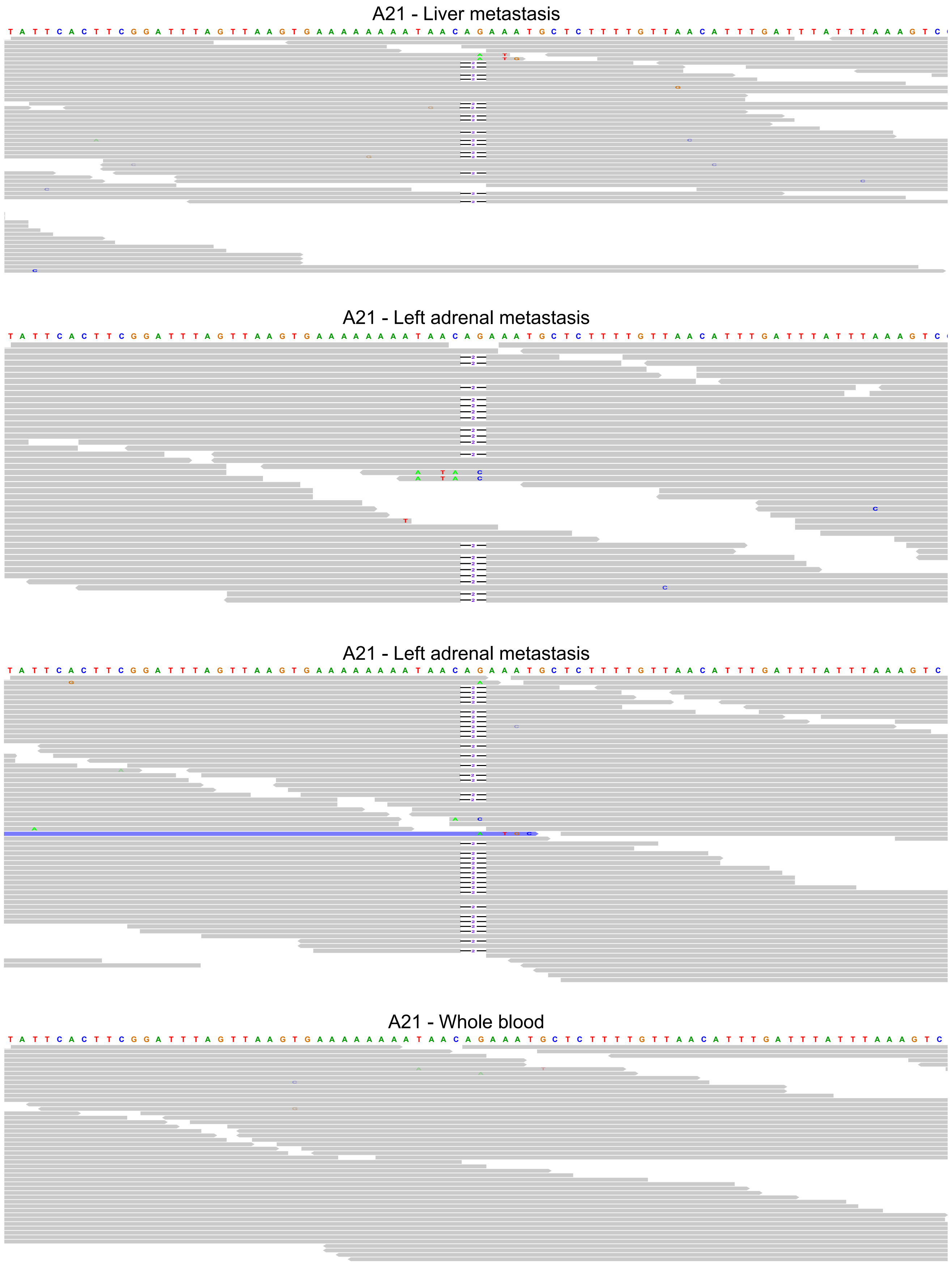

Supplementary Figure 6. Integrative Genomics Viewer (IGV) visualization of the reads supporting a somatic FOXA1 3'-UTR mutation in metastatic tissues of patient A21.

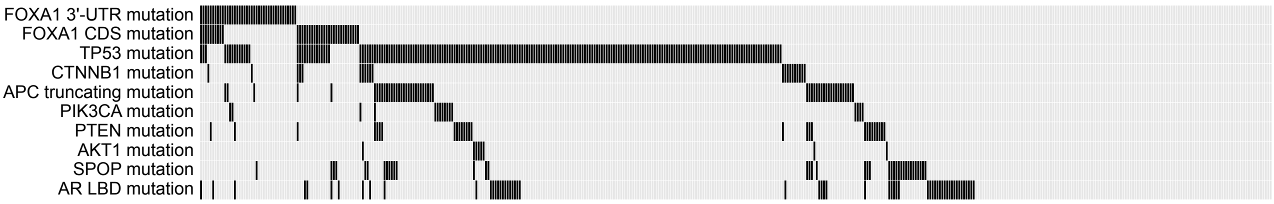

	P-value for co-occurrence with FOXA1 3'-UTR mutation
FOXA1 CDS mutation	**0.001**
TP53 mutation	0.21
CTNNB1 mutation	1.00
APC truncating mutation	0.63
PIK3CA mutation	0.86
PTEN mutation	1.00
AKT1 mutation	0.93
SPOP mutation	0.24
AR LBD mutation	0.48

	P-value for co-occurrence with any FOXA1 mutation
TP53 mutation	0.66
CTNNB1 mutation	0.38
APC truncating mutation	0.42
PIK3CA mutation	1.00
PTEN mutation	0.76
AKT1 mutation	0.55
SPOP mutation	0.61
AR LBD mutation	0.81

Supplementary Figure 7. Oncoprint showing co-occurrence of *FOXA1* 3'-UTR mutations with recurrent mutations in other genes. Tables below the oncoprint show P-values for co-occurrence with *FOXA1* 3'-UTR mutations and all *FOXA1* mutations. P-values were calculated using Fisher's exact test.

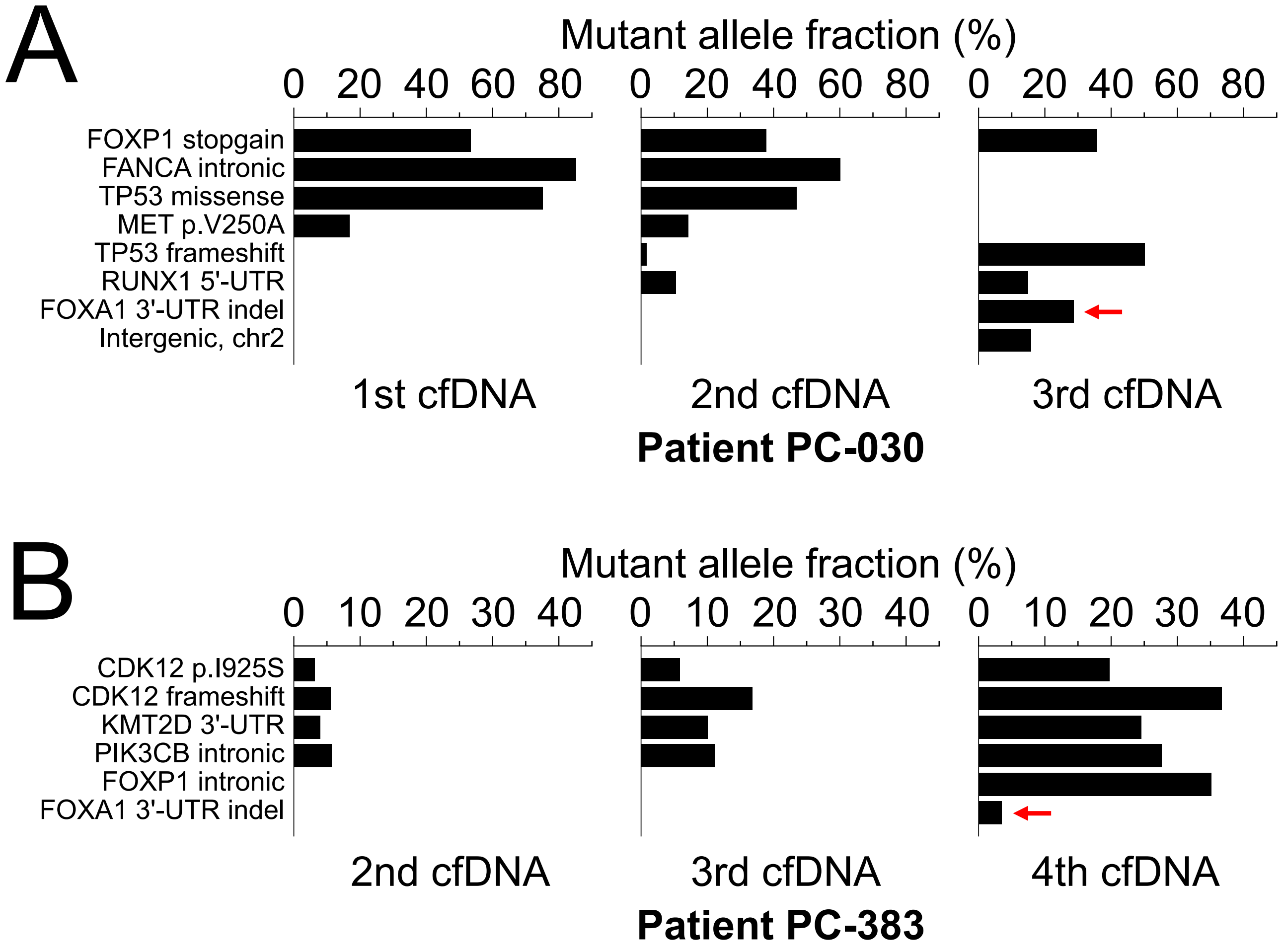

Supplementary Figure 8. Barplot showing allele fractions of somatic mutations in cfDNA samples from patients PC-030 and PC-383. Both patients displayed evidence for late emergence of a FOXA1 3'-UTR mutation harboring clone. (**A**) In patient PC-030, there was evidence for a significant clonal switch between the 2nd and 3rd cfDNA timepoints, accompanied by the emergence of a FOXA1 3'-UTR mutation. (**B**) In patient PC-383, the FOXA1 3'-UTR indel appeared the 4th cfDNA timepoint together with a FOXP1 intron mutation, suggesting a clonal expansion.

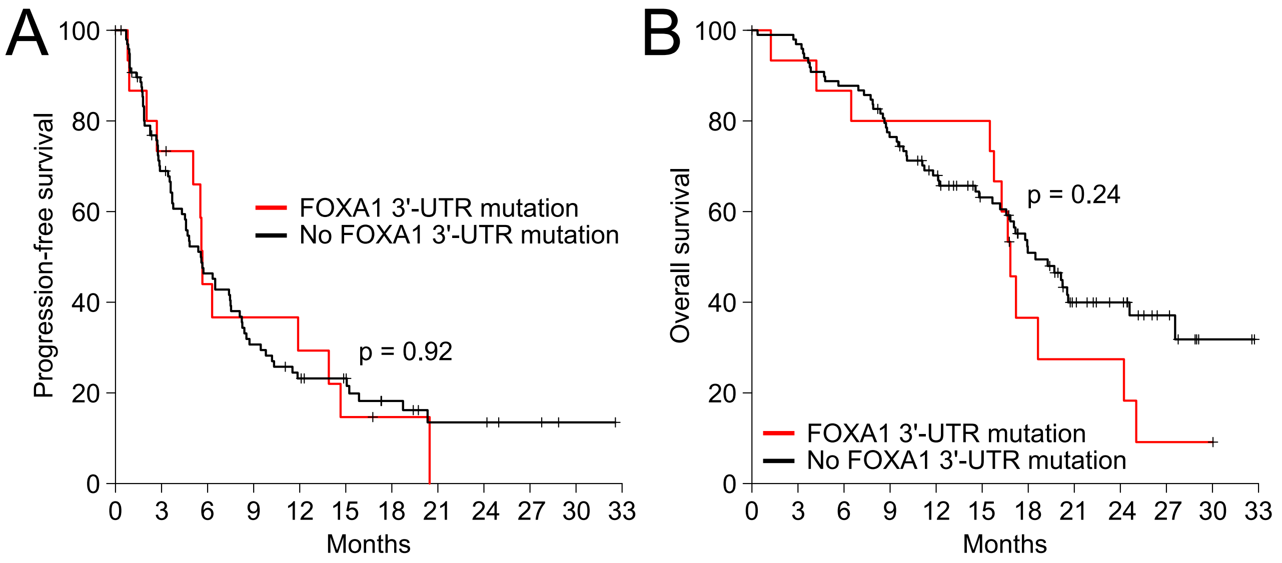

Supplementary Figure 9. Analysis for association between *FOXA1* 3'-UTR mutations and clinical outcome. (**A**) Kaplan-Meier estimates of progression-free survival for patients with and without *FOXA1* 3'-UTR mutations, in a cohort of 202 mCRPC patients treated with first-line abiraterone or enzalutamide. (**B**) Kaplan-Meier estimates of overall survival for patients with and without *FOXA1* 3'-UTR mutations, in a cohort of 202 mCRPC patients treated with first-line abiraterone or enzalutamide.

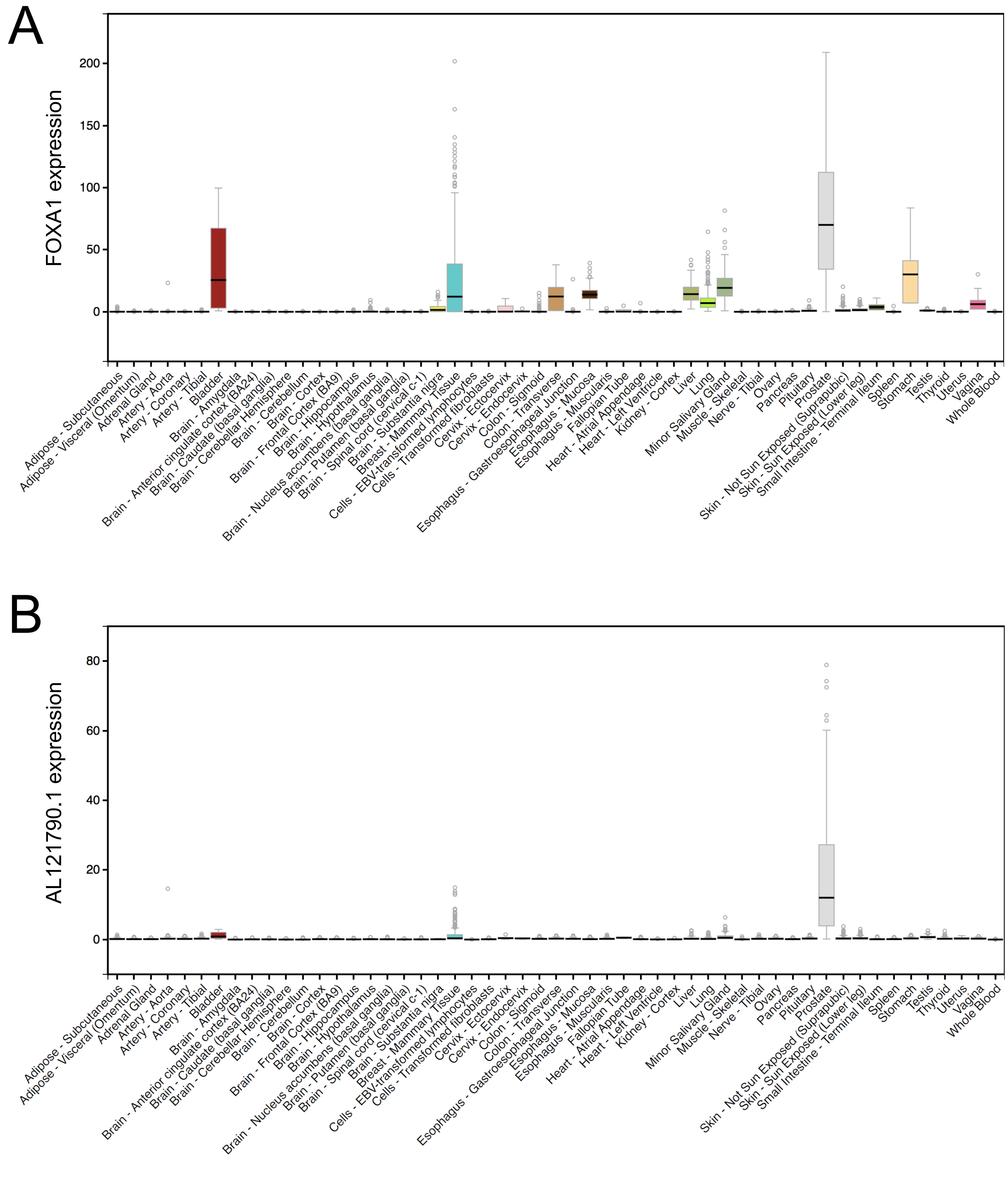

Supplementary Figure 10. *FOXA1* and *AL121790.1* are only expressed in some adult tissues. The expression of *FOXA1* and *AL121790.1* is highest in prostate tissue, although expression of both is also seen in breast and bladder tissues. (**A**) *FOXA1* expression boxplots for Genotype-Tissue Expression (GTEx) project tissue types. (**B**) *AL121790.1* expression boxplots for GTEx tissue types.

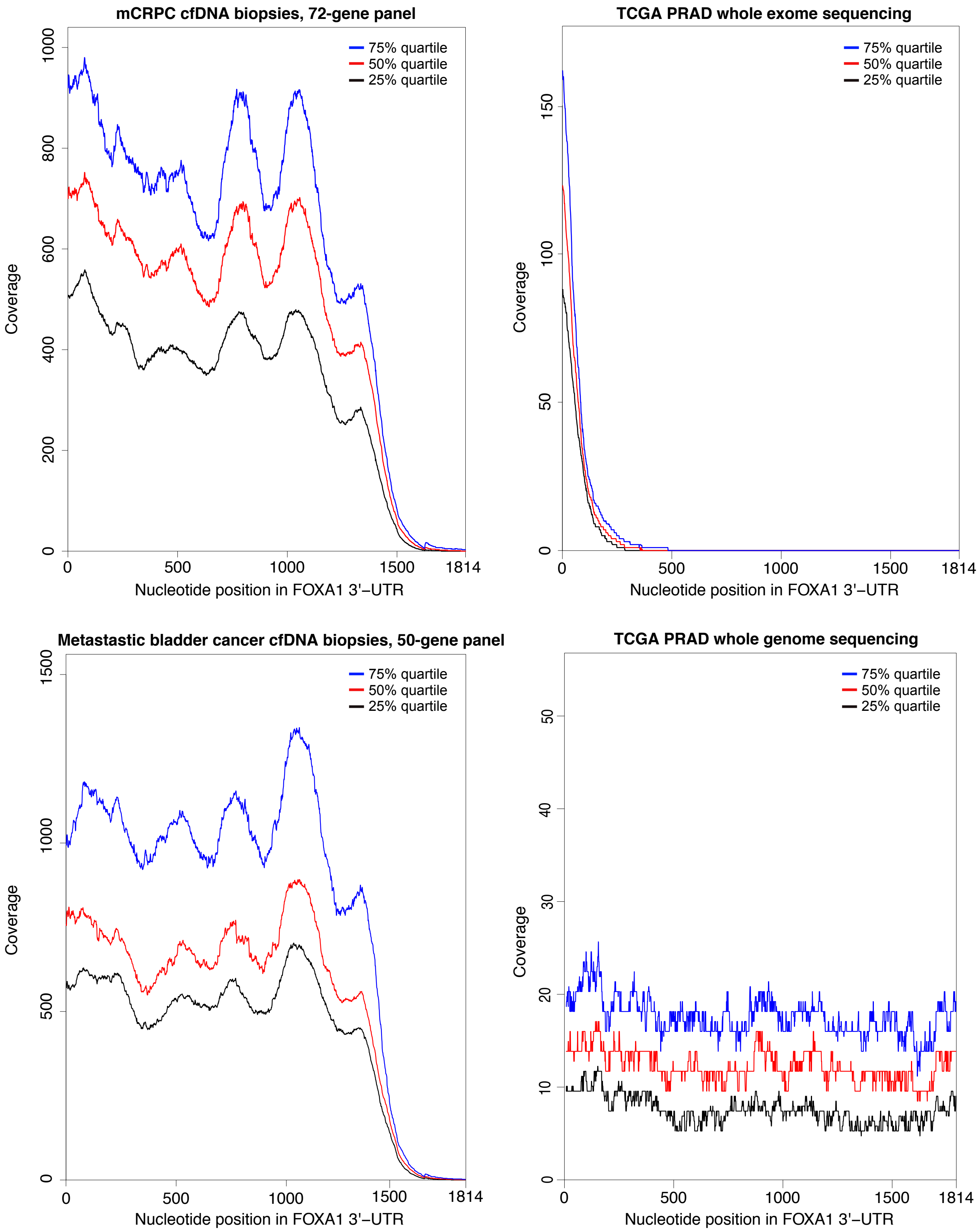

Supplementary Figure 11. Comparison of the sequencing coverage attained within the FOXA1 3'-UTR in four different datasets: our mCRPC cfDNA sequencing cohort (72-gene targeted panel), our metastatic bladder cancer cfDNA sequencing cohort (50-gene targeted panel), TCGA prostate adenocarcinoma whole exome sequencing cohort, and TCGA prostate adenocarcinoma whole genome sequencing cohort. High sequencing coverage of the FOXA1 3'-UTR was achieved by in both cfDNA cohorts. Whole exome panels do not capture the 3'-UTR.

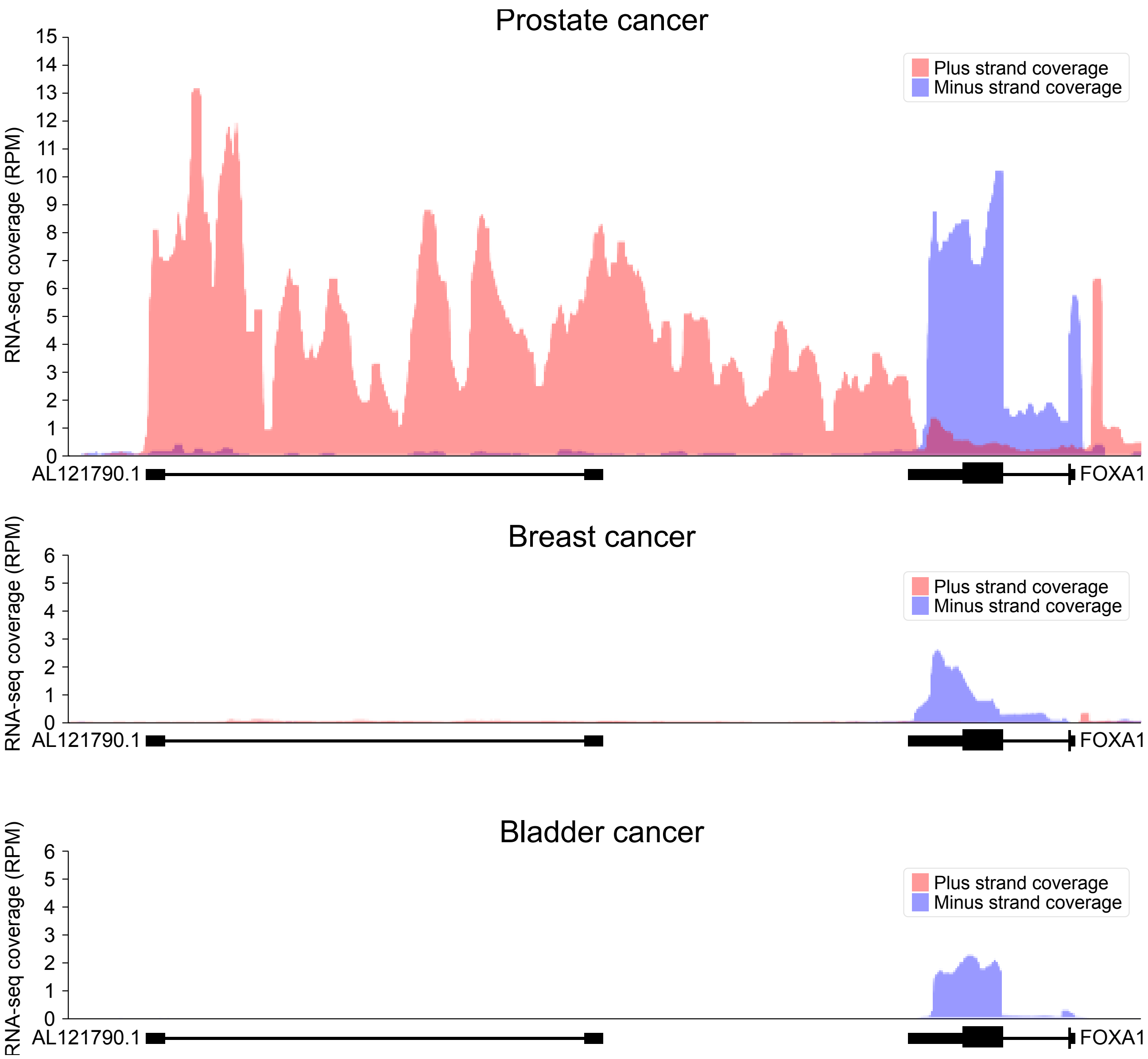

Supplementary Figure 12. Strand-specific RNA-seq coverage of the *FOXA1* and *AL121790.1* locus in cancers of the prostate, breast and bladder. Evidence for strong *AL121790.1* expression, and bidirectional transcription at the FOXA1 3'-UTR, was only observed in prostate cancer. RNA-seq coverage was normalized by library size (reads per million, RPM).
