# Supplementary material for: Frequent mutation of the FOXA1 untranslated region in prostate cancer
Source: Commun Biol. 2018 Aug 24;1:122. doi: 10.1038/s42003-018-0128-1 (PMC6123809; doi:10.1038/s42003-018-0128-1)
Supplement: Supplementary file 2 — Description of Additional Supplementary Information [file 42003_2018_128_MOESM2_ESM.docx]

**Description of Additional Supplementary Files**

File Name: Supplementary Data 1

Description: All 712 metastatic prostate cancer cfDNA samples analyzed as part of this study. Sequencing coverage, circulating tumor DNA fraction, and somatic mutation count is shown for every sample.

File Name: Supplementary Data 2

Description: List of genes included in the 72-gene capture panel. For each gene, the dominant splice variant and length of the annotated coding and untranslated regions is shown. Number of bases with high sequencing coverage (100x or higher) in each region is also shown.

File Name: Supplementary Data 3

Description: List of all annotated coding and untranslated regions for the 72 genes. Number of bases with high sequencing coverage (100x or higher) for each region is also shown.

File Name: Supplementary Data 4

Description: All somatic UTR mutations detected in the 439 circulating tumor DNA positive blood samples from 290 metastatic prostate cancer patients.

File Name: Supplementary Data 5

Description: Somatic FOXA1 3’-UTR mutations identified in tumor tissue.

File Name: Supplementary Data 6

Description: Adjacent DNA sequence neighborhood of FOXA1 3’-UTR mutations. Rightmost column indicates which indels were considered to represent microsatellite instability.

File Name: Supplementary Data 7

Description: Clinical followup data for the 201 patients that participated in the published abiraterone-enzalutamide trial cohort. Patient 125 from the trial was excluded due to lack of sequenced cfDNA samples.
